# Supplementation of carnitine leads to an activation of the IGF-1/PI3K/Akt signalling pathway and down regulates the E3 ligase MuRF1 in skeletal muscle of rats

**DOI:** 10.1186/1743-7075-10-28

**Published:** 2013-03-15

**Authors:** Janine Keller, Aline Couturier, Melanie Haferkamp, Erika Most, Klaus Eder

**Affiliations:** 1 Institute of Animal Nutrition and Nutritional Physiology, Justus-Liebig-University, Heinrich-Buff-Ring 26-32, Giessen, 35392, Germany

**Keywords:** Carnitine, Rat, Muscle, Ubiquitin-proteasome system, IGF-1/PI3K/Akt signalling pathway

## Abstract

**Background:**

Recently, it has been shown that carnitine down-regulates genes involved in the ubiquitin-proteasome system (UPS) in muscle of pigs and rats. The mechanisms underlying this observation are yet unknown. Based on the previous finding that carnitine increases plasma IGF-1 concentration, we investigated the hypothesis that carnitine down-regulates genes of the UPS by modulation of the of the IGF-1/PI3K/Akt signalling pathway which is an important regulator of UPS activity in muscle.

**Methods:**

Male Sprague–Dawley rats, aged four weeks, were fed either a control diet with a low native carnitine concentration or the same diet supplemented with carnitine (1250 mg/kg diet) for four weeks. Components of the UPS and IGF-1/PI3K/Akt signalling pathway in skeletal muscle were examined.

**Results:**

Rats fed the diet supplemented with carnitine had lower mRNA and protein levels of MuRF1, the most important E3 ubiquitin ligase in muscle, decreased concentrations of ubiquitin-protein conjugates in skeletal muscle and higher IGF-1 concentration in plasma than control rats (P < 0.05). Moreover, in skeletal muscle of rats fed the diet supplemented with carnitine there was an activation of the PI3K/Akt signalling pathway, as indicated by increased protein levels of phosphorylated (activated) Akt1 (P < 0.05).

**Conclusion:**

The present study shows that supplementation of carnitine markedly decreases the expression of MuRF1 and concentrations of ubiquitinated proteins in skeletal muscle of rats, indicating a diminished degradation of myofibrillar proteins by the UPS. The study moreover shows that supplementation of carnitine leads to an activation of the IGF-1/PI3K/Akt signalling pathway which in turn might contribute to the observed down-regulation of MuRF1 and muscle protein ubiquitination.

## Background

The protein turnover represents a finely regulated balance between protein synthesis and degradation. Many regulatory proteins have very short half-time and are rapidly hydrolyzed to their constituent amino acids and replaced by newly synthesized proteins. In mammalian tissues, proteins are degraded mainly by the ubiquitin-proteasome system (UPS) [1,2]. The UPS is part of all eukaryotic cells and involves a complex cascade of enzymes and displays a high degree of specificity towards its numerous substrates [3]. The tagging process, which involves the linking of ubiquitin chain to proteins via an isopeptide bond, is the rate limiting process mediated by three enzymatic steps. First, ubiquitin is bound via its carboxyl terminus to the sulfhydryl group of ubiquitin-activating enzyme (E1). Thereafter, ubiquitin is transferred from E1 to ubiquitin-conjugating enzyme (E2), via formation a high energy thioester linkage [4]. In the last step, the specific ubiquitin-ligase enzymes (E3), in skeletal muscle mainly muscle RING finger-1 (MuRF1) and atrogin-1 (MAFbx, muscle atrophy F-box), catalyzes the transfer of ubiquitin from E2 to the ε-amino group of the target protein. Polyubiquitinated proteins can be recognized by the downstream 26S proteasome, a multicatalytic protease complex, and rapidly degraded into smaller peptides [5,6]. The expression of MuRF1 and atrogin-1 is markedly up-regulated during several catabolic states such as cancer, kidney failure, burn injury, sepsis, muscle denervation, diabetes and starvation [7-10]. It was shown that genetic deletion of MuRF1 and atrogin-1 results in significantly increased sparing of muscle mass loss induced by muscle denervation. This clearly demonstrates the important role of these atrophy-regulated genes as key factors of myofibrillar protein breakdown by the ubiquitin-dependent proteolysis [7].

With regard to the regulation of the UPS pathway, it was shown that elevated insulin-like growth factor 1 (IGF-1) levels lead to a reduction in the expression of MuRF1 and atrogin-1 and, thereby, a decreased protein degradation by the 26S proteasome in skeletal muscle [11]. Binding of IGF-1 to its receptor induces a conformational change in the IGF-1 receptor tyrosine kinase, resulting in a multiple auto-phosphorylation cascade. As a consequence, phosphoinositide-3-kinase (PI3K) is activated and downstream Akt (also named protein kinase B) translocates to the membrane, where it becomes phosphorylated (at threonine^308^ and serine^473^) and thereby activated by PI3K [12,13]. Activation of PI3K/Akt in turn leads to phosphorylation and inactivation of a specific transcription factor family named forkhead box O transcription factors (FoxOs). Consequently, the translocation of FoxOs into the cell nucleus is repressed and the induction of transcription of the skeletal muscle-specific E3 ligases, atrogin-1 and MuRF1 is not initiated [14].

Recently, it has been shown that supplementation of carnitine (3-hydroxy-4-N,N,N-trimethylaminobutyric acid), an endogenous compound best known for its function in the transport of long-chain fatty acids into the mitochondrion, leads to a down regulation of genes involved in the UPS in skeletal muscle of growing pigs [15]. More recently, it has been found that carnitine even inhibits proteasomal protein degradation and reduces muscle wasting in a rat model of cancer cachexia [16]. Thus, carnitine could be a promising compound to suppress muscle wasting under catabolic conditions. However, the mechanism underlying the effect of carnitine on the expression of genes of the UPS is yet unknown. In myotubes, used as a model of skeletal muscle cells, incubation with carnitine did not influence the expression of genes of the UPS, suggesting that carnitine has no direct but rather indirect effects on the UPS in muscle cells [15]. Studies in animals and humans have shown that supplementation of carnitine increases plasma concentrations of IGF-1 [17-19]. Due to the important role of IGF-1 in the activation of the IGF-1/PI3K/Akt signalling pathway, we hypothesized that the effect of carnitine on the UPS in skeletal muscle could be mediated by an activation of the IGF-1/PI3K/Akt signalling pathway. To investigate this hypothesis, we performed an experiment with rats, fed either a control diet or a diet supplemented with carnitine, and considered UPS and the IGF-1/PI3K/Akt signalling pathway in skeletal muscle.

## Methods

### Animal experiment

Twenty-four male Sprague–Dawley rats obtained from Charles River (Sulzfeld, Germany) at four weeks of age with an average body weight of 111 (± 4 SD) g were housed in groups of three animals in Macrolon cages in a room controlled for temperature (22 ± 1°C), relative humidity (50–60%), and light (12-h-light/-dark cycle). After seven days of acclimatization, rats were randomly assigned to two groups (n = 12/group) and individually housed. A basal diet with a low carnitine concentration (< 1 mg/kg diet) was formulated. This diet consisted of (g/kg): Wheat (310), barley (156), corn (150), oats (50), extracted soybean meal (250), soy oil (30), L-lysine (9), DL-methionine (5), mineral mixture (30), vitamin mixture (10). Vegetable diet components were used as sources of protein as they have lower concentrations of carnitine than protein sources of animal origin. This diet contained 14.4 MJ metabolizable energy and 194 g crude protein per kg. Concentrations of amino acids, minerals and vitamins were in agreement with the requirements for growing rats, according to the AIN-93G reference [20]. The control group received this diet without carnitine supplementation, whereas the “carnitine group” received the diet supplemented with 1250 mg L-carnitine/kg (obtained from Lohmann Animal Health, Cuxhaven, Germany). Food and water was available ad libitum during the whole experiment. The diets were fed for 28 days. All experimental procedures were in strict accordance with the recommendations in the guidelines for the care and use of laboratory animals [21] and the Appendix A of European Convention for the Protection of Vertebrate Animals used for Experimental and other Scientific Purposes. In Accordance with Article 4 par. 3 of the German Animal Welfare Law all animals were humanely killed for scientific purpose approved by the Animal Welfare Officer of the Justus-Liebig-University, JLU No. 422_M.

### Sample collection

At day 29, rats were killed by decapitation under anesthesia with carbon dioxide. Blood was collected in heparinized tubes and plasma was obtained by centrifugation (1,100 g; 10 min; 4°C). Liver and skeletal muscle (*M*. *quadriceps femoris*, *M*. *extensor digitorum longus*, *M*. *gastrocnemius*, *M*. *soleus*) samples were excised. All muscles from both the left and right limb were weighed and subsequently snap-frozen in liquid nitrogen. All tissue samples were stored at −80°C pending further analysis.

### Determination of concentrations of protein and triglycerides in muscles

For determination of the protein concentration, frozen muscle samples (30 mg) were homogenized in RIPA buffer (radioimmunoprecipitation assay buffer; 50 mM Tris, 150 mM NaCl, 10% glycerol, 0.1% SDS, 1% Triton X-100, 1 mM EDTA, 0.5% deoxycholate, 1% protease inhibitor mix; pH 7.5) using an Ultraturrax (IKA Werke GmbH, Staufen, Germany). The homogenate was centrifuged at 16,200 *g* (4°C) for 15 min. Protein concentrations were determined in the supernatants using the bicinchoninic acid protein assay kit (Interchim, Montluçon, France) with BSA as standard.

Lipids from muscles were extracted with a mixture of n-hexane and isopropanol (3:2, vol/vol) [22]. For lipid analyses, aliquots of the lipid extracts were dried and the lipids were dissolved using a 1:1-mixture of chloroform and Triton X-100. Subsequently, concentrations of triglycerides in samples were determined by an enzymatic reagent kit (Fluitest TG, obtained from Analyticon Biotechnologies AG, Lichtenfels, Germany) [23].

### Carnitine analysis

Concentrations of free carnitine and acetyl carnitine in plasma and tissues were determined by tandem mass spectrometry with deuterated carnitine-d3 (Cambridge Isotype Laboratories, Andover, MA, USA) as internal standard as described recently in detail [24]. Total carnitine was calculated as the sum of free carnitine and acetyl carnitine.

### RNA isolation and Quantitative real-time RT-PCR (qPCR)

Total RNA was isolated from 20 mg of each liver - and samples of *M*. *quadriceps femoris* using Trizol reagent (Invitrogen, Karlsruhe, Germany) according to the manufacturer’s protocol. Genomic DNA was removed from total RNA isolated with on-column DNase I digestion using RNeasy Mini Kit columns (Qiagen, Germany). After RNA isolation, concentration and purity were estimated from the optical density at 260 and 280 nm, respectively, using an Infinite 200M microplate reader and a NanoQuant Plate (both from Tecan, Männedorf, Switzerland). The integrity of the total RNA was checked by 1% agarose gel electrophoresis. RNA was judged as suitable only if the samples exhibited intact bands corresponding to the 18S and 28S ribosomal RNA subunits. Isolated RNA was preserved at −80°C until use. Relative mRNA concentrations were determined using real-time detection RT-PCR (qPCR) carried out on a Rotorgene 2000 system (Corbett Research, Mortlake, Australia). For this end, cDNA was synthesized from 1.2 μg of total RNA using 100 pmol dT18 primer (Eurofins MWG Operon, Ebersberg, Germany), 1.25 μL 10 mmol/L dNTP mix (GeneCraft, Lüdinghausen, Germany), 5 μl buffer (Fermentas, St. Leon-Rot, Deutschland), and 60 units M-MuLV Reverse Transcriptase (MBI Fermentas, St. Leon-Rot, Germany) at 42°C for 60 min, and a final inactivating step at 70°C for 10 min in Biometra Thermal Cycler (Whatman Biometra®, Göttingen, Germany). Subsequently, cDNA was stored in aliquots at −20°C. For the standard curve a cDNA pool of all samples each from liver and muscle was made. qPCR was performed using 2 μL cDNA combined with 18 μL of a mixture composed of 10 μl KAPA SYBR FAST qPCR Universal Mastermix (Peqlab, Erlangen, Germany), 0.4 μL each of 10 μM forward and reverse primers and 7.2 μL DNase/RNase free water in 0.1 mL tubes (Ltf Labortechnik, Wasserburg, Germany). Gene-specific primer pairs obtained from Eurofins MWG Operon were designed using PRIMER3 and BLAST. Characteristics of primer pairs are listed in Table 1. Wherever possible, matching primers were designed to be located in different exons. The amplification of single product of the expected size was approved using 2% agarose gel electrophoresis stained with GelRed™ nucleic acid gel stain (Biotium Inc., Hayward, CA). To minimize the influence of confounding variables and to improve the accuracy of the quantification process, expression values of target genes were normalized using the GeNorm normalization factor. GeNorm is a freely available software based on the excel platform and allows the selection of the most eligible reference gene by using the geometric mean of the expression of the candidate cDNA [25]. In order to calculate the normalization factor, all Ct values were transformed into relative quantification data by using the 2^-△Ct^ equation [26], and the highest relative quantities for each gene were set to 1, as described previously [15]. From these values the GeNorm normalization factor was calculated from the three reference genes showing the lowest *M* values (liver: CANX, YWHAZ, MDH1; muscle: CANX, YWHAZ, TOP1; Table 1), indicating the most stable expression out of the six tested candidate reference genes. Candidate reference genes are usually chosen from housekeeping genes which are not regulated or influenced by the experimental procedure. Six housekeeping genes were selected from commonly used reference genes listed by the company primerdesign (URL: http://www.primerdesign.co.uk/genorm_all_species.html). After normalization of gene expression data using the calculated GeNorm normalization factor, means and SD were calculated from normalized expression data for samples of the same treatment group. The mean of the control group was set to 1 and means and SD of the treatment group were scaled proportionally. Data on qPCR performance for each gene measured in liver and muscle are shown in Table 1.

**Table 1:** **Characteristics and performance data of the primers used for reference gene**-**stability measure *M *and qPCR**

**Gene**	**Forward primer (5**^**′**^**→3**^**′**^**)**	**Product size (bp)**	**NCBI GenBank**	**Slope**		**R2**^**1**^		**Efficiency**^**2**^		***M***	
	**Reverse primer (5**^**′**^**→3**^**′**^**)**			**Liver**	**Muscle**	**Liver**	**Muscle**	**Liver**	**Muscle**	**Liver**	**Muscle**
*Reference genes*											
ATP5B	GCACCGTCAGAACTATTGCT	203	NM_134364.1	−0.31	−0.34	0.99	0.99	2.04	2.00	0.069	0.149
	GAATTCAGGAGCCTCAGCAT										
CANX	CCAGATGCAGATCTGAAGAC	175	NM_172008.2	−0.32	−0.32	0.99	0.99	2.10	1.98	0.065	0.082
	CTGGGTCCTCAATTTCACGT										
MDH1	CAGACAAAGAAGAGGTTGCC	206	NM_033235.1	−0.32	−0.32	0.99	0.99	2.07	2.10	0.054	0.111
	CGTCAGGCAGTTTGTATTGG										
RPL13	CTTAAATTGGCCACGCAGCT	198	NM_031101.1	−0.23	−0.30	0.88	0.99	1.70	2.01	0.072	0.105
	CTTCTCAACGTCTTGCTCTG										
TOP1	GAAGAACGCTATCCAGAAGG	137	NM_022615.1	−0.29	−0.30	0.99	0.99	1.95	2.20	0.066	0.088
	GCTTTGGGACTCAGCTTCAT										
YWHAZ	GACGGAAGGTGCTGAGAAA	198	NM_013011.3	−0.30	−0.30	0.98	0.99	2.02	2.08	0.057	0.082
	GCAGCAACCTCAGCCAAGT										
*Target genes*											
IGF-1	CCCGGGACGTACCAAAATGAGCG	354	NM_001082477.2	−0.29		0.99		1.97			
	ATGTCAGTGTGGCGCTGGGC										
FBXO32	GACTGGACTTCTCGACTGCC	242	NM_133521.1		−0.29		0.98		1.94		
	GACTTGCCGACTCTCTGGAC										
TRIM63	AAGGCAGCCACCCGATGTGC	110	NM_080903.1		−0.31		0.99		2.02		
	GCCTGGTGAGCCCCGAACAC										

^1^Coefficient of determination of the standard curve.

^2^The efficiency was determined by [10^-slope^].

ATP5B: ATP synthase, H+ transporting, mitochondrial F1 complex, beta polypeptide; CANX: calnexin; MDH1: soluble malate dehydrogenase; RPL13: ribosomal protein L13; TOP1: topoisomerase (DNA) I; YWHAZ: tyrosine 3-monooxygenase/tryptophan 5-monooxygenase activation protein, zeta polypeptide; IGF-1: insulin-like growth factor 1; FBXO32: F-box protein 32; TRIM63: tripartite motif containing 63.

### Immunoblot analysis

Tissue homogenates and subsequent protein determination in samples of *M*. *quadriceps femoris* were carried out as described above. For immunoblot analysis, 30 μg protein from each homogenate were separated on 12.5% SDS-PAGE and electrotransferred to a nitrocellulose membrane (Pall Corporation, Pensacola, FL, USA). To detect ubiquitin-protein conjugates 30 μg protein from each homogenate were separated on 7.5% SDS-PAGE. Loading of equal amounts of protein in each line was verified by Ponceau S (Carl Roth, Karlsruhe, Germany) staining. After incubation the membranes overnight at 4°C in blocking solution (5% nonfat dried milk powder), membranes were incubated with primary antibodies against MuRF1 (polyclonal anti-MuRF1 antibody; Santa Cruz Biotechnology, Inc., Santa Cruz, Ca, USA), atrogin-1/Fbx32 (polyclonal anti-Fbx32 antibody; Abcam, Cambridge, UK), ubiquitin (polyclonal anti-ubiquitin antibody, Abcam, Cambridge, UK), Akt1 (polyclonal anti-Akt1 antibody; Cell Signaling Technology, Boston, MA, USA), hosphor-Akt1 (at Ser^473^) (monoclonal anti-pAkt1 antibody; Cell Signaling Technology, Boston, MA, USA), FoxO1 (polyclonal anti-FoxO1 antibody; Cell Signaling Technology, Boston, MA, USA), hosphor-FoxO1 (at Ser^256^) (polyclonal anti-pFoxO1 antibody; Cell Signaling Technology, Boston, MA, USA), mTOR (polyclonal anti-mTOR antibody; Cell Signaling Technology, Boston, MA, USA), hosphor-mTOR (at Ser^2481^) (polyclonal anti-pmTOR antibody; Cell Signaling Technology, Boston, MA, USA), hosphor-mTOR (at Ser^2448^) (polyclonal anti-pmTOR antibody; Cell Signaling Technology, Boston, MA, USA) and α-Tubulin (polyclonal anti-α-Tubulin antibody; Cell Signaling Technology, Boston, MA, USA) and GAPDH (monoclonal anti-GAPDH antibody; Abcam, Cambridge, UK) as reference proteins to control for adequate normalization. The membranes were washed, and then incubated with a horseradish peroxidase conjugated secondary monoclonal anti-mouse-IgG antibody (Sigma-Aldrich, Steinheim, Germany) for hosphor-Akt1, polyclonal anti-rabbit-IgG antibody (Sigma-Aldrich, Steinheim, Germany) for atrogin-1/Fbx32, ubiquitin, Akt1, FoxO1, hosphor-FoxO1, α-Tubulin, mTOR, phosphor-mTOR (Ser^2448^ and Ser^2481^) and polyclonal anti-goat-IgG antibody (Santa Cruz Biotechnology, Santa Cruz, Ca, USA) for MuRF1 at RT. Afterwards blots were developed using ECL Plus or ECL Advanced (both GE Healthcare, Munich, Germany). The signal intensities of specific bands were detected with a Bio-Imaging system (Syngene, Cambridge, UK) and quantified using Syngene GeneTools software (nonlinear dynamics).

### Determination of IGF-1

The concentration of IGF-1 in plasma of rats was determined using a commercial enzymelinked immunosorbent assay (ELISA) kit (R&D Systems, Wiesbaden-Nordenstadt, Germany).

### Statistical analysis

Values presented in the text are means ± SD. Data were analyzed by one factorial analysis of variance with dietary carnitine concentration as factor using the Minitab statistical software (Release 13, Minitab Inc., State College, PA, USA). Statistical significance of differences of the mean values of the two groups of rats was evaluated using Student’s *t* test. Means were considered significantly different at P < 0.05.

## Results

### Growth performance and skeletal muscle weights

Food intake, final body weights after four weeks, feed conversion ratio (g feed/g body weight gain) and weights of *M*. *quadriceps femoris*, *M*. *extensor digitorum longus*, *M*. *gastrocnemius* and *M*. *soleus* were not different between both groups of rats (P > 0.05; Table 2).

**Table 2:** **Growth performance data**, **skeletal muscle weights**, **muscle protein concentrations and muscle triglyceride concentrations of rats fed diets without** (**Control**) **or with supplemented carnitine** (**Carnitine**)

	**Control**	**Carnitine**
**Food intake (g/d)**	26.3 ± 2.4	25.8 ± 2.1
**Final body weight (g)**	353 ± 31	334 ± 28
**Feed conversion ratio (g feed/g BWG)**	3.11 ± 0.54	3.20 ± 0.44
**Muscle weights (g)**		
*M*. *quadriceps femoris*	1.08 ± 0.08	1.09 ± 0.07
*M*. *EDL*	0.51 ± 0.07	0.52 ± 0.09
*M*. *gastrocnemius*	1.86 ± 0.09	1.90 ± 0.08
*M*. *soleus*	0.15 ± 0.01	0.15 ± 0.02
**Muscle protein concentration (mg/g wet tissue)**		
*M*. *quadriceps femoris*	108 ± 10	111± 11
*M*. *EDL*	102 ± 9	113± 10^*^
*M*. *gastrocnemius*	108 ± 15	111± 15
**Muscle triglyceride concentration (mg/g wet tissue)**		
*M*. *quadriceps femoris*	1.42 ± 0.66	1.28 ± 0.46
*M*. *EDL*	1.06 ± 0.43	0.84 ± 0.17
*M*. *gastrocnemius*	1.29 ± 0.32	1.24 ± 0.33

Values are means ± SD (n = 12/group). ^*^ indicates a significant difference to the control group (P < 0.05). BWG: body weight gain; M: *musculus*; EDL: *extensor digitorum longus*.

### Concentrations of proteins and triglycerides in muscles

In order to investigate whether carnitine supplementation could have an influence on the composition of muscles, concentrations of protein and triglycerides in *M*. *quadriceps femoris*, *M*. *extensor digitorum longus and M*. *gastrocnemius* were determined. Protein concentrations in *M*. *quadriceps femoris* and *M*. *gastrocnemius* did not differ between the two groups of rats, while rats fed the diet supplemented with carnitine had a higher protein concentration in *M*. *extensor digitorum longus* than rats of the control group (P < 0.05, Table 2). Concentrations of triglycerides did not differ in any muscle between the two groups of rats (P > 0.05; Table 2). Total masses of protein in the three muscles as well as the ratios between protein and triglycerides in the three muscles considered did not differ between the two groups (data not shown).

### Carnitine concentrations in plasma, liver and skeletal muscle

Rats fed the diet supplemented with carnitine had higher concentrations of free carnitine, acetyl carnitine and total carnitine in plasma, liver and muscle than control rats (P < 0.05; Table 3).

**Table 3:** **Concentrations of free carnitine**, **acetyl carnitine and total carnitine in plasma**, **liver and muscle of rats fed diets without** (**Control**) **or with supplemented carnitine** (**Carnitine**)

	**Control**	**Carnitine**
**Plasma (μmol/l)**		
Free carnitine	36 ± 5	91 ± 7^*^
Acetyl carnitine	16 ± 3	42 ± 6^*^
Total carnitine	52 ± 7	133 ± 10^*^
**Liver (nmol/g dry matter)**		
Free carnitine	595 ± 70	1142 ± 158^*^
Acetyl carnitine	23 ± 13	74 ± 25^*^
Total carnitine	618 ± 72	1216 ± 142^*^
**Muscle (nmol/g dry matter)**		
Free carnitine	2153 ± 321	3465 ± 420^*^
Acetyl carnitine	1246 ± 198	2571 ± 266^*^
Total carnitine	3399 ± 452	6036 ± 450^*^

Values are means ± SD (n = 12/group). ^*^ indicates a significant difference to the control group (P < 0.05).

### Relative mRNA and protein concentrations of FBXO32 (encoding atrogin-1) and TRIM63 (encoding MuRF1) in skeletal muscle

**Figure 1:**
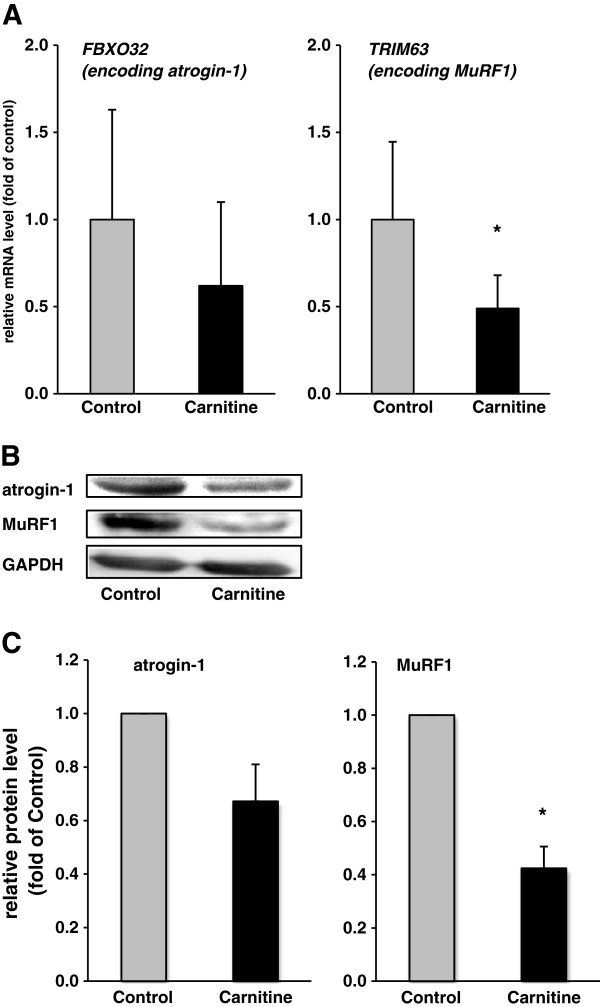
**Relative mRNA abundance of *FBXO32 *and TRIM63 (A) and relative protein concentrations of atrogin**-**1 and MuRF1 (B,C) in *M***. ***quadriceps femoris *of rats fed either a control diet (0 mg carnitine/kg diet**; **Control**) **or a diet supplemented with 1250 mg carnitine/kg diet (Carnitine).** (**A**) Bars are means ± SD (n = 12/group). The normalized expression ratio in the control group is set to 1.0. ^*^ indicates a significant difference to the control group (P < 0.05). (**B**) Representative immunoblots specific to atrogin-1, MuRF1 and GAPDH as internal control are shown for one animal per group; immunoblots for the other animals revealed similar results. (**C**) Bars represent data from densitometric analysis and represent means ± SD (n = 6/group); bars are expressed relative to the protein level of the control group (= 1.00). ^*^ indicates a significant difference to the control group (P < 0.05).

Rats fed the diet supplemented with carnitine had lower relative mRNA and protein concentrations of tripartite motif containing 63 (TRIM63; encoding MuRF1) in the muscle than control rats (P < 0.05; Figure 1A-C). Relative mRNA- and protein concentrations of F-box protein 32 (FBXO32; encoding atrogin-1) in muscle were slightly lower in rats fed the diet supplemented with carnitine than in control rats; the differences were however not statistically significant (Figure 1A-C).

### Relative protein concentrations of ubiquitinated protein conjugates

**Figure 2:**
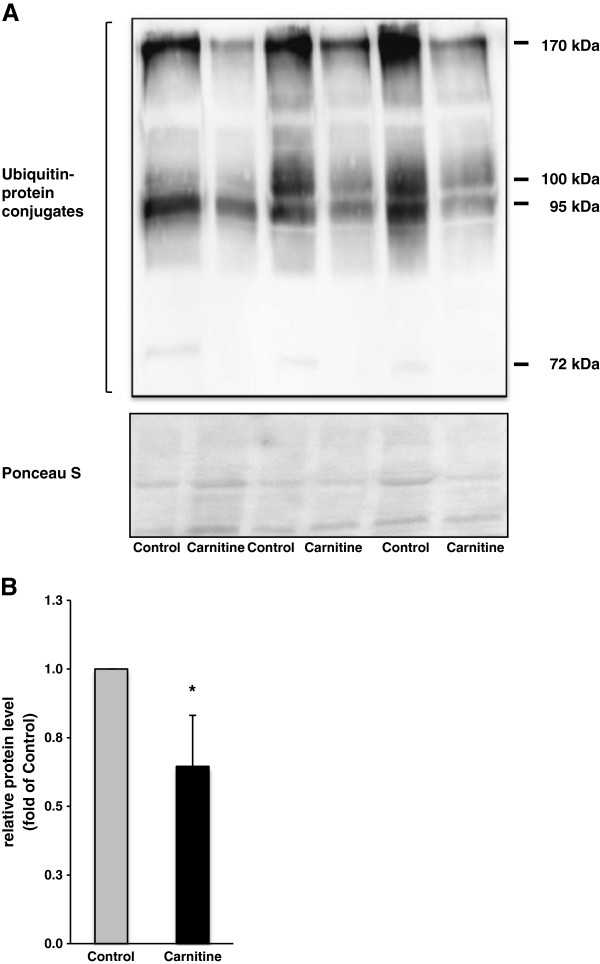
**Relative protein levels of ubiquitin**-**protein conjugates in *M. quadriceps femoris *of rats fed either a control diet (0 mg carnitine/kg diet; Control) or a diet supplemented with 1250 mg carnitine/kg diet (Carnitine).** (**A**) A representative immunoblot specific to ubiquitin is shown for three animals per group; immunoblots for the other animals revealed similar results. Reversible staining of nitrocellulose membranes with Ponceau S revealed equal loading of protein. (**B**) Bars represent data from densitometric analysis and represent means ± SD (n = 6/group); bars are expressed relative to the protein level of the control group (= 1.00). ^*^ indicates a significant difference to the control group (P < 0.05).

In line with the decreased expression of MuRF1, rats fed the diet supplemented with carnitine had lower amounts of ubiquitinated proteins with different molecular masses in skeletal muscle than control rats (P < 0.05; Figure 2).

### Relative mRNA concentration of IGF-1 in liver and plasma concentration of IGF-1

**Figure 3:**
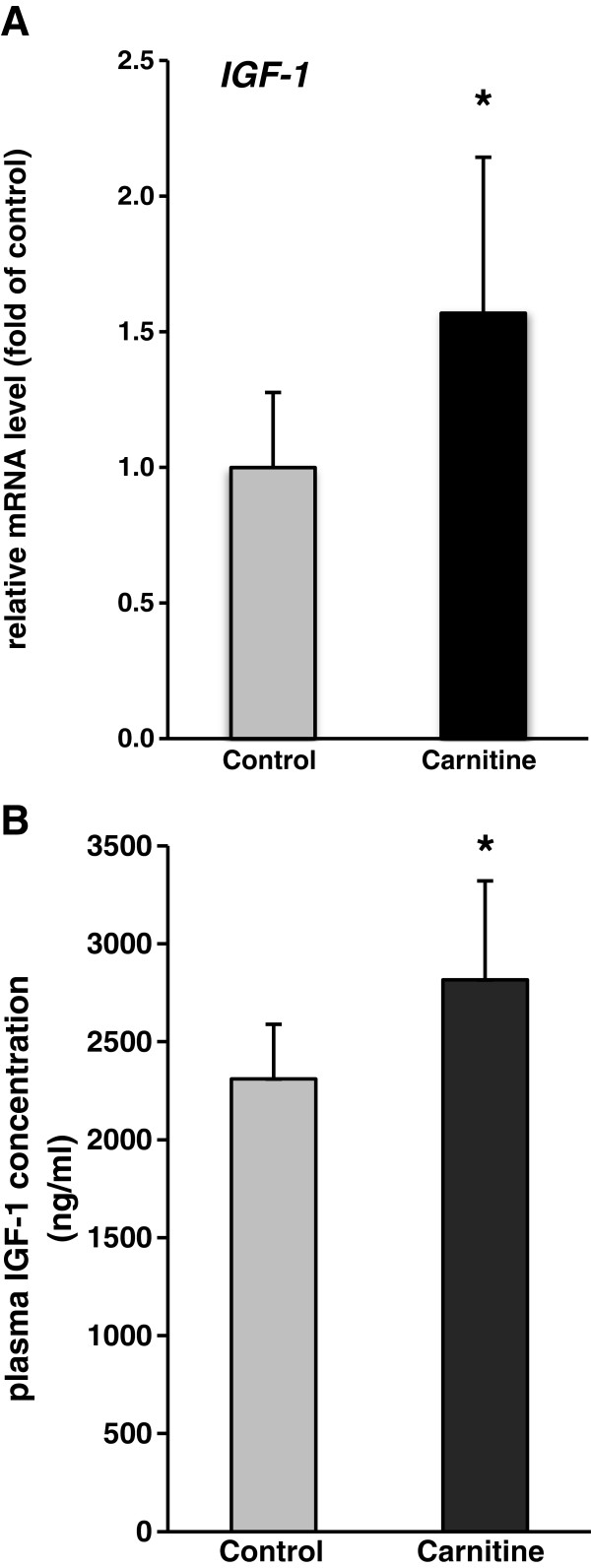
**(A) Relative mRNA abundance of *IGF***-***1 *in liver of rats fed either a control diet (0 mg carnitine/kg diet**; **Control) or a diet supplemented with 1250 mg carnitine/kg diet (Carnitine).** Bars are means ± SD (n = 12/group). The normalized expression ratio in the control group is set to 1.0. ^*^ indicates a significant difference to the control group (P < 0.05). (**B**) Concentration of IGF-1 (ng/ml) in plasma of rats fed either a control diet (0 mg carnitine/kg diet; Control) or a diet supplemented with 1250 mg carnitine/kg diet (Carnitine). Bars represent mean ± SD (n = 12/group). ^*^ indicates a significant difference to the control group (P < 0.05).

Rats fed the diet supplemented with carnitine had higher relative mRNA concentrations of IGF-1 in the liver and a higher concentration of IGF-1 in plasma than control rats (P < 0.05; Figure 3A and B).

### Relative protein concentrations of total and phosphorylated Akt1, FoxO1 and mTOR

**Figure 4:**
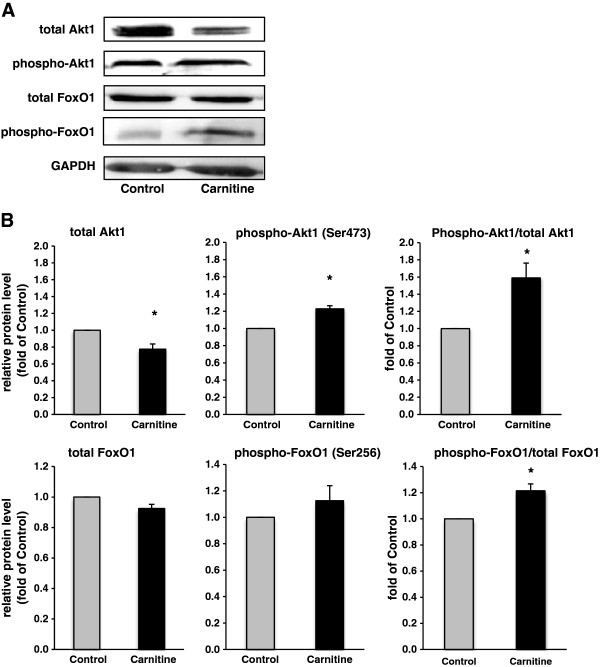
**Relative protein concentrations of total and phosphorylated Akt1 and FoxO1 and calculated phospho**-**Akt1/total Akt1 and phospho-FoxO1/total FoxO1 ratios in *M. quadriceps femoris *of rats fed either a control diet (0 mg carnitine/kg diet; Control) or a diet supplemented with 1250 mg carnitine/kg diet (Carnitine).** (**A**) Representative immunoblots specific to total Akt1, phospho-Akt1, total FoxO1, phospho-FoxO1 and GAPDH as internal control are shown for one animal per group; immunoblots for the other animals revealed similar results. (**B**) Bars represent data from densitometric analysis and represent means ± SD (n = 6/group); bars are expressed relative to the protein level of the control group (= 1.00). ^*^ indicates a significant difference to the control group (P < 0.05).

**Figure 5:**
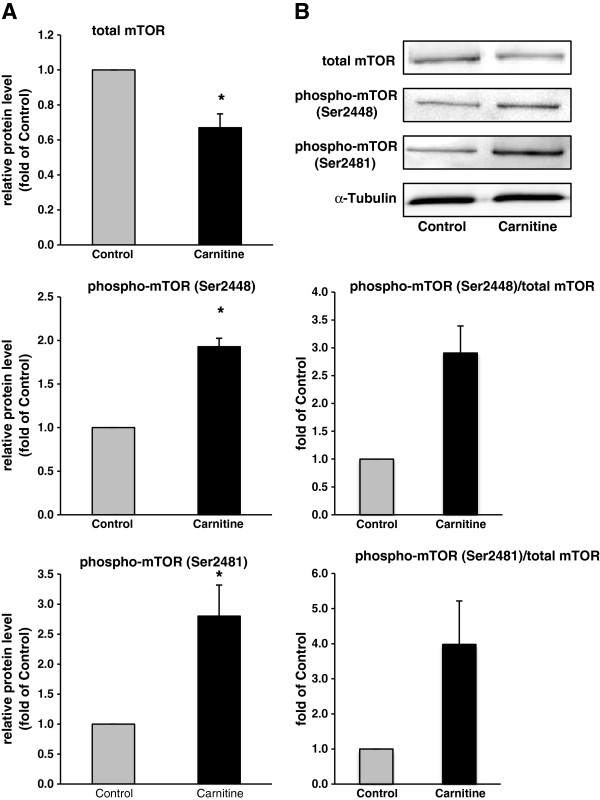
**Relative protein concentrations of total and phosphorylated mTOR at Ser**^**2448 **^**and Ser**^**2481 **^**and calculated phospho**-**mTOR (Ser**^**2448**^**)/total mTOR and phosphor**-**mTOR (Ser**^**2481**^**)/total mTOR ratios in *M. quadriceps femoris *of rats fed either a control diet (0 mg carnitine/kg diet; Control) or a diet supplemented with 1250 mg carnitine/kg diet (Carnitine).** (**A**) Representative immunoblots specific to total mTOR, phosphor-mTOR (Ser^2448^), phosphor-mTOR (Ser^2481^) and α-Tubulin as internal control are shown for one animal per group; immunoblots for the other animals revealed similar results. (**B**) Bars represent data from densitometric analysis and represent means ± SD (n = 6/group); bars are expressed relative to the protein level of the control group (= 1.00). ^*^ indicates a significant difference to the control group (P < 0.05).

Rats fed the diet supplemented with carnitine had a reduced concentration of total Akt1, an increased concentration of phospho-Akt1 concentration and an increased ratio of phospho-Akt1 to total Akt1 in skeletal muscle (P < 0.05; Figure 4A and B), indicative of an activation of IGF-1/PI3K/Akt signalling pathway. The protein concentration of total and phosphorylated FoxO1 in skeletal muscle was not different between both groups of rats (Figure 4A and B). However, the ratio of phospho-FoxO1 to total FoxO1 was lower in skeletal muscle of rats fed the diet supplemented with carnitine than in control rats, indicating that carnitine stimulates phosphorylation and thereby inactivation of FoxO1 (P < 0.05; Figure 4A and B). As activation of IGF-1/PI3K/Akt signalling pathway is known to stimulate protein synthesis by activation of the mammalian target of rapamycin (mTOR), we also determined protein levels of total and phospho-mTOR (at Ser^2448^ and Ser^2481^) and calculated the phospho-mTOR and total mTOR ratio. The protein concentration of total mTOR in skeletal muscle was reduced in rats fed the diet supplemented with carnitine whereas the concentrations of phospho-mTOR (Ser^2448^, Ser^2481^) were markedly increased compared to control rats (P < 0.05; Figure 5A and B). The ratios of phospho-mTOR (Ser^2448^ and Ser^2481^) to total mTOR tended to be higher in skeletal muscle of rats fed the diet supplemented with carnitine compared to control rats (P < 0.1; Figure 5A and B).

## Discussion

Recent studies have shown that supplementation of carnitine suppresses the expression of genes of the UPS in skeletal muscle and reduces loss of muscle mass under catabolic conditions [15,16]. The present study investigated the hypothesis that this phenomenon could be mediated by an activation of the IGF-1/PI3K/Akt signalling pathway. To investigate this hypothesis, we performed a feeding experiment with rats fed either a control diet with a low native carnitine concentration or the control diet supplemented with 1250 mg carnitine per kg, a dose which – according to the recent experiments [15,16] - might be effective to induce the expected effect on genes of the UPS. The finding that supplementation of carnitine does not influence body weight gains of the growing rats but markedly increases plasma and tissue carnitine concentrations agrees with several other studies [15,27-29]. In this study, we found that supplementation of carnitine suppresses the expression of MuRF1 in skeletal muscle but does not significantly reduce the expression of atrogin-1. This finding agrees with the recent study of Busquets et al. [16] showing that carnitine lowers the expression of MuRF1 but not that of atrogin-1 in skeletal muscle of rats suffering from cancer cachexia. In contrast to these findings in rats, supplementation of carnitine in pigs lowered the expression of both of the E3 ligases [15], indicating that the effect of carnitine on the expression of E3 ligases in skeletal muscle might be species-specific. In agreement with a down-regulation of MuRF1, we observed reduced concentrations of ubiquitin-protein conjugates in skeletal muscle of rats fed a diet supplemented with carnitine. For technical reasons, we did not examine muscle protein breakdown in this study. Ubiquitination is a rate-limiting step in the proteolysis of muscle proteins by the UPS [30]. Therefore it seems likely that protein degradation in muscle was also reduced by carnitine in rat skeletal muscle. It has been shown that myofibrillar proteins, like myosin light chain proteins are the main targets of MuRF1 for ubiquitination [31]. Thus, carnitine might suppress particularly the degradation of myofibrillar proteins, which under physiological conditions comprise around 60% of total muscle proteins [31]. In contrast to MuRF1, atrogin-1 tags primarily proteins for degradation which are important for controlling protein synthesis and myoblast differentiation, like myogenic factor MyoD, myogenin and the eukaryotic initiation factor of protein synthesis eIF3-f [32-35].

The present study confirms recent studies in humans, pigs, rats and chickens in showing that supplementation of carnitine increases plasma IGF-1 concentrations [17-19,36-38]. IGF-1 is an important regulator of the activity of the UPS. Several studies have shown that IGF-1 inhibits muscle protein degradation [39-42]. This effect is induced by an activation of the PI3/Akt signalling pathway which in turn inhibits the expression of E3 ligases due to an inactivation of FoxOs [14,43]. In the present study, we indeed observed an activation of the PI3/Akt signalling pathway and an inactivation of FoxOs in the skeletal muscle of rats fed a diet supplemented with carnitine. These observations suggest that down-regulation of MuRF1 in muscle of rats fed a diet supplemented with carnitine was indeed mediated by increased plasma concentrations of IGF-1 and an activation of the PI3/Akt signalling pathway. It is known that IGF-1 induces muscle hypertrophy not only by suppression of protein degradation but also by an increase in protein synthesis due to activation of the downstream mTOR-pathway [44]. According with this function of IGF-1, we observed an increase in phosphorylated mTOR at Ser^2448^ and Ser^2481^ in skeletal muscle of rats fed the diet supplemented with carnitine compared to control rats. Although we did not study protein synthesis in muscle, this finding suggests that carnitine could also promote protein synthesis in skeletal muscle.

Down-regulation of E3-ligases in muscle and an activation of the mTOR-pathway are expected to increase skeletal muscle weights and to improve whole body N-balance. Indeed, in the study of Busquets et al. [16], suppression of the UPS by carnitine led to a reduction of muscle wasting and to an improvement of the whole body N-balance in rats suffering from cancer cachexia. Unexpectedly, although carnitine exerted clear effects on UPS and mTOR-pathway via an increase of plasma IGF-1 concentrations, no changes in body weights and muscle weights of the rats were observed in the present study. This finding agrees with a recent study in fast growing pigs, in which carnitine supplementation did also not improve weight gains and the feed conversion ratio in spite of a clear down-regulation of E3-ligases in liver and skeletal muscle [15]. In pigs, it has been however observed that supplementation of carnitine enhances protein accretion at the expense of fat deposition [45,46]. In order to investigate whether carnitine could have changed the composition of muscle, we determined protein and triglyceride concentrations in skeletal muscles. We did however not find differences in the protein/triglyceride ratios, suggesting that carnitine did also not change composition of skeletal muscle in healthy, growing rats. In growing animals, the rate of protein synthesis strongly exceeds protein degradation [47]. What we suspect is that under the condition of fast growth, the effect of suppression of the UPS by carnitine on muscle protein balance might be superimposed by the high rate of protein synthesis. Probably, the suppression of the UPS by carnitine with respect to muscle protein degradation might be more pronounced and more relevant under condition of negative N balance, chronic diseases and denervation or under unloading conditions. Under such conditions, the UPS is strongly up-regulated leading to muscle atrophy and a reduction of the mass of skeletal muscles [7,48,49]. The finding of Busquets et al. [16] that carnitine reduces muscle wasting in rats suffering from cancer cachexia indeed suggests that carnitine exerts beneficial effects on muscle mass particularly under conditions associated with an increased activity of the UPS.

In conclusion, the present study shows that supplementation of carnitine to rats leads to a down-regulation of MuRF1 and a decrease in the content of ubiquitinated proteins in skeletal muscle, indicating that degradation of myofibrillar proteins by the UPS was reduced. The study moreover suggest that this effect could be at least in part due to increased plasma levels of IGF-1 which in turn led to an activation of the IGF-1/PI3K/Akt signalling pathway and to an inactivation of FoxO1, the transcriptional regulator of MuRF1.

## Competing interests

The authors declare that they have no competing interests.

## Authors’ contributions

JK and KE designed research and coordinated the study; JK and MH carried out the feeding experiment; JK and AC carried out the molecular biological analyses; MH and EM carried out the carnitine analyses; JK and KE wrote the paper. KE had primary responsibility for final content. All authors read and approved the final manuscript.
